# Evolution of control with learning classifier systems

**DOI:** 10.1007/s41109-018-0088-x

**Published:** 2018-08-13

**Authors:** Matthew R. Karlsen, Sotiris Moschoyiannis

**Affiliations:** Department of Computer Science, Faculty of Engineering and Physical Sciences, University of Surrey, Guildford, GU2 7XH Surrey UK

**Keywords:** Controllability, Learning, Discovery, Boolean network, Intervention, Complex systems, LCS, XCS

## Abstract

In this paper we describe the application of a learning classifier system (LCS) variant known as the eXtended classifier system (XCS) to evolve a set of ‘control rules’ for a number of Boolean network instances. We show that (1) it is possible to take the system to an attractor, from any given state, by applying a set of ‘control rules’ consisting of ternary conditions strings (i.e. each condition component in the rule has three possible states; 0, 1 or #) with associated bit-flip actions, and (2) that it is possible to discover such rules using an evolutionary approach via the application of a learning classifier system. The proposed approach builds on learning (reinforcement learning) and discovery (a genetic algorithm) and therefore the series of interventions for controlling the network are determined but are not fixed. System control rules evolve in such a way that they mirror both the structure and dynamics of the system, without having ‘direct’ access to either.

## Introduction

Complexity theory has been applied to the study of a number of different fields including biology (Kauffman 1993), economics (Anderson et al. 1988), technology studies (Frenken 2006) and digital ecosystems (Krause et al. 2009). Achieving control over a system or systems, through intervention(s) to reach a particular state, is often a major goal in such endeavours. The challenge of achieving control is non-trivial due to complex interdependencies arising from the interplay between system components arranged in a myriad of different potential architectures.

In many of the real world systems modelled, there is a need or desire to intervene and control the system (Cornelius et al. 2013; Moschoyiannis et al. 2016), to bring about a particular state or constrain operation to a particular attractor of the system. A number of approaches to the question of control have been developed. Some of these approaches focus on the structure of the network, determining the *driver* nodes (Liu et al. 2011) that exert more influence on the system than other nodes and hence are best targeted by an intervention to the system. The application of control theory is an example of such an approach (Liu et al. 2011; Moschoyiannis et al. 2016) and a web-based tool *CCTool* (available at: http://cctool.herokuapp.com) has been developed to aid in identifying strategic intervention points (Moschoyiannis et al. 2016). Other approaches instantiate a model of the network and aim to understand how the dynamics of the system affects its controllability, e.g., see (Cornelius et al. 2013; Kim et al. 2013). For example Cornelius et al. (2013) control models of power networks and also a network model of cancer emergence in humans. Similarly, Kim et al. (2013) model control of ‘biomolecular regulatory networks’. This is the approach we take in this paper.

A key characteristic of a complex system is that it continuously evolves, e.g., due to dynamic changes in the roles, states and behaviours of the entities involved. In an attempt to harness the arising complexity, work in this direction has targeted the graph topology of the network reflecting the structure of the modelled system, e.g. (Liu et al. 2012; Haghighi and Namazi 2015; Savvopoulos et al. 2017). In previous work (Savvopoulos and Moschoyiannis 2017) we have also focused on structure and studied the effect of removing nodes (and edges) on the minimal sets of *control* or *driver* nodes.

In this paper we consider control in relation to both network structure and dynamics. We are concerned with the application of Learning Classifier Systems (LCSs) (Urbanowicz and Moore 2009) that are able to control Boolean networks (Kauffman 1969). More specifically, we focus on using the LCS variant ‘XCS’ (Wilson 1995; 1998) to control random Boolean networks of the NK type (Kauffman 1993). We select NK Boolean networks due to them being both comparatively simple to understand and easy to construct, whilst at the same time possessing a state comprised of the variable settings of individual nodes. Whilst clearly simpler than many real world networks due to their Boolean nature, the approach herein can be generalised to regulate both integer-based and real-valued variables through the XCS extensions XCSI (Wilson 2000b) and XCSR (Wilson 2000a).

Random boolean networks (RBNs), as developed and communicated by Kauffman (1969, 1989, 1993) and others, consist of simple processing units assembled with a directed graph structure. Each unit takes one or more binary inputs and supplies one or more binary outputs. Each node is connected to other nodes via directed links indicating the direction in which binary signals are transmitted. The precise relationship between the inputs and outputs of a particular processing unit is determined by a randomly created logic function. The ‘wiring up’ of the processing units is also randomly determined. The resulting random networks assembled in this way exhibit complex behaviour, with a large state space, many possible trajectories through this space, and multiple basins of attraction. The precise graph topology of a random Boolean network graph is determined by the random generation process. NK Boolean networks consist of N nodes each with K inputs selected from other randomly determined nodes in the network.

Random Boolean networks are closely associated with specific Boolean networks constructed to model real world systems. Boolean networks that model real-world systems are identified and used within the work of Kim et al. (2013) and include cell cycle networks, gene regulatory networks (GRNs), protein interaction networks, and cell signalling networks. Gates et al. (2016) analyse a number of Boolean network models for real-world systems including the ‘Albert and Othmer segment polarity network’. Kauffman et al. (2003) specifically consider RBNs in relation to the ‘yeast transcriptional network’. Giacomantonio and Goodhill (2010) apply RBN modelling to the gene regulatory network that determines ‘mammalian cortical area development’. Through RBN application they explore all possible network behaviours for the genes in question, selecting the subset of networks that produce the behaviours observed in an experimental setting. Common network features were then identified in the subset, providing more information on the unknowns within the actual network. Though we apply the approach developed herein on random Boolean networks it may also be applicable to these specific Boolean network models for real-world systems.

LCSs (Urbanowicz and Moore 2009) are rule-based machine learning techniques that comprise a learning element (reinforcement) and a discovery element – typically, Genetic Algorithms (GAs) are applied. The objective is to determine a set of rules that collectively capture and apply knowledge in order to make predictions. In our work, we apply an adaptation of the XCS learning classifier system (Wilson 1995; 1998) to evolve a set of rules that when applied take the system being modelled as an RBN from any given state to a target basin of attraction. XCS has been selected because its accuracy-based fitness tends to provide superior performance to earlier LCS systems (Wilson 1995). That it is a well-established LCS variant with a substantial supporting literature also counts in its favour (see the number of XCS and variant papers cited within the LCS review by Urbanowicz and Moore (2009)).

We apply reinforcement learning to this *controllability problem* so that if a response is desirable then rules leading to actions that led to that state are ‘strengthened’. Over time, rules that lead to a basin of attraction, in the particular network, are more likely to be triggered whilst those that have proven unfit are gradually eliminated. It is in this sense that we talk about *evolving rule sets* and hence the *evolution of control* in our approach.

The work herein illustrates how XCS can be used as a controller for a simple network. However, we believe that the overall approach may be used to control more complex real world networks. Wherever a network has Boolean, integer or real-valued variables that can be manipulated or influenced in some way, directly or indirectly, an eXtended Classifier System variant may be constructed that endeavours to ‘learn’ and control the network.

Our approach differs to others in a number of ways. First, an LCS intervention can potentially consist of a number of small ‘nudges’, where appropriate, rather than a single large intervention. Second, LCS rules use #-symbol ‘don’t care’ wildcards in addition to 0 and 1 values for direct matching. These wild cards mean that you need far fewer rules than the number of states in the system to cover the full state space. The resulting ternary (three state) classifier rules mean that the LCS is able to cover large areas of the state space in a compact form. Third, when considering dynamic networks, it is possible that an LCS will be able to continuously adapt to a network subject to external perturbations rather than proving brittle to such changes. Finally, it is worth noting that the state of a Boolean network can be most easily represented as a bit string – this naturally fits with the ternary structure of the LCS rules.

One other contribution may be clearly stated. The application of XCS in the way we have proposed learns to control a system via the creation of internal knowledge (i.e. the classifier rules) *from scratch*. No human knowledge is required inside the XCS itself (though the detectors must be configured correctly for XCS to function).

The remainder of this paper is organised as follows. “Random Boolean networks” Section outlines random Boolean networks while “Learning classifier systems” Section presents key characteristics of learning classifier systems. “Using LCS rule sets to control RBNs” Section introduces the idea of using LCS rule sets to control RBNs. “Constructing XCS” Section describes the construction of the particular XCS used in our approach and the post-rule processing optimisation performed, whilst “Selection of XCS parameters” Section provides an overview of setting the parameters. A selection of experiments to demonstrate the approach are described in “Experiments” Section. The results are analysed in “Results and Discussion” Section which also includes the limitations of the approach and aspects that deserve further investigation. Related work is discussed in “Related work” Section but also throughout the text. The paper finishes in “Concluding remarks” Section with concluding remarks.

## Random Boolean networks

Random Boolean networks were introduced by Kauffman in (Kauffman 1969), with later refinements in (Kauffman 1989; 1993). Kauffman specifically developed the idea of NK Boolean Networks (not to be confused with the NK model of tunably rugged fitness landscapes described elsewhere (Kauffman and Levin 1987; Kauffman and Weinberger 1989)) where the overall structure of the network is governed by two parameters, N and K. Here N is the number of nodes in the network whilst K is the number of inputs to each node from randomly selected nodes in the network. The directed links terminating at a particular node may thus potentially include a link emerging from the node in question, forming a loop. In the remainder of this section we outline Random Boolean Networks, drawing from the descriptions provided by Kauffman in (Kauffman 1989; 1993) unless otherwise noted.

To construct an NK Boolean Network, N nodes are first created. For each node, K nodes are randomly selected and a directed link is created from each of the K nodes to the current node in question. For each of the N nodes a random logic function is created and assigned to the node. When *K*=2 this logic function may well be one of the well known Boolean functions such as AND and OR, *though this is not definite*. Each node has a binary state (0 or 1) which is randomly selected at the time of model creation and then updated as the model runs.

The network dynamics take place in a number of discrete time steps. The precise behaviour depends upon whether the updates are performed asynchronously (one update per time step) or synchronously (all nodes updated at each time step). The binary state of each node is updated based on the state of the input nodes connected to that node. For an example we shall use an ‘OR node’. For a given set of two inputs, an OR node will output a 1 if either or both input values are 1 (it will otherwise output a 0). So, for instance, if the OR node finds one of its input nodes is set to 1 whilst the other is set to 0, then its state will be switched to 1 in the next time instant (the current state of the node is irrelevant, except as a potential input to other nodes). With synchronous updating the results of each update must clearly be ‘cached’ temporarily and then assigned to the nodes once all of the update calculations have been completed.

Whilst the construction of an NK boolean network is extremely simple, the behaviour of the network can be varied and complex (Kauffman 1993): the states of the network form a directed graph of their own, with a sequence of states forming a ‘trajectory’ through the state space of the system. Eventually the system settles in to a ‘state cycle’ – the attractor of the system – where a small number of states repeat in sequence. The state space can thus be seen to be divided in to ‘basins of attraction’ where all systems with states in a specific basin ‘flow’ towards the attractor of the basin. For more information on RBN behaviour, please refer to the work of Kauffman (1989, 1993). A comprehensive tutorial on Random Boolean Networks has been produced by Aldana et al. (2003).

**Fig. 1:**
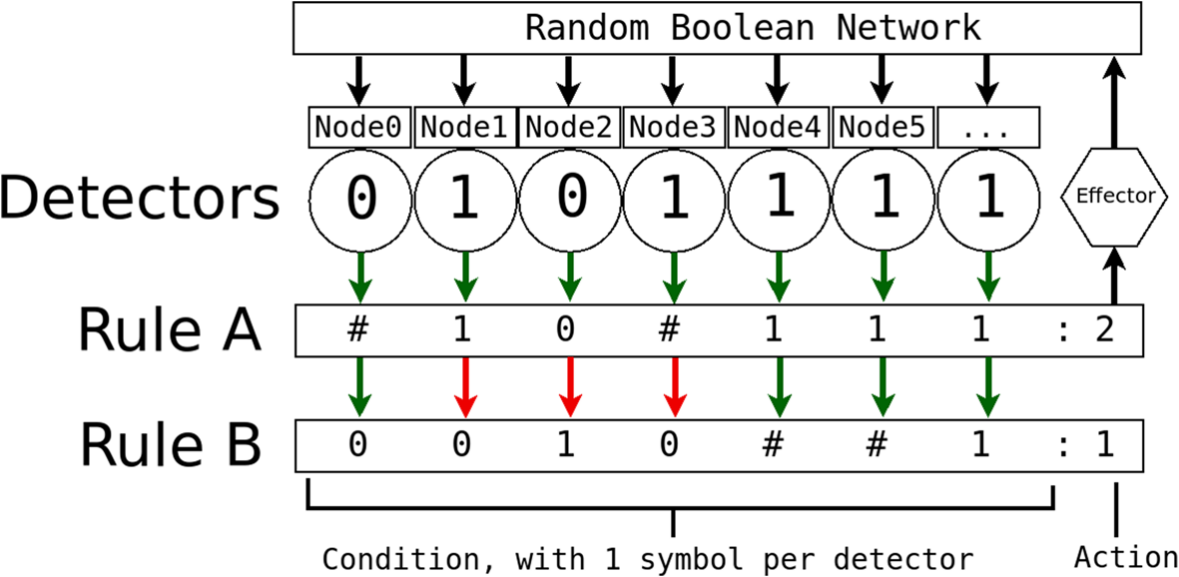
A simple example of rule matching with two rules with ternary (three state) condition components and one binary environmental state. Adapted from (Matthew R Karlsen and Sotiris Moschoyiannis: Learning condition action rules for personalised journey recommendations, forthcoming)

Previous work has considered control in the context of the dynamics of the system (Cornelius et al. 2013). In the context of control we can see that the challenge of achieving a given state depends upon both the current position (within one of the system’s basins of attraction) and also the target state Fig. 1 (Cornelius et al. 2013). One may wish to accelerate reaching the state cycle of a particular basin from within that basin. Alternatively one may wish to shift the system from a state within one basin of attraction to an attractor within another basin – a more complex feat. Finally, one may wish to achieve a non-attractor state – an intervention that would require constant maintenance.

## Learning classifier systems

Learning classifier systems (Urbanowicz and Moore 2009) are rule-based machine learning techniques that detect the state of the environment and then take actions on the environment, in response to this state, with the goal of achieving a reward. Internally the LCS contains a number of condition–action rules, a subset of which may be triggered by each environmental state (see Fig. 1). The response to an action is assessed via a ‘reinforcement program’. If the response is desirable, the rules leading to the actions that led to that state are ‘strengthened’ by increasing an indicator of quality associated with those rules (the precise mechanism used varies across learning classifier systems). Rule variety and improvement is introduced in to the system via a genetic algorithm. Over time, the rules that lead to desirable system states are more likely to be triggered whilst those that have proven unfit are eliminated – the system has learned to interact with its environment to bring about a desirable environmental state or states.

A minimal classifier system functions as follows (Urbanowicz and Moore 2009). Firstly a ‘detector’ converts the state of the environment in to some form of internal representation (usually a bit string). This bit string is compared with a population of rules. Each rule consists of a ternary string, an associated action, and a fitness. The ternary strings consist of 0s, 1s and # (‘don’t care’) symbols. (A detector and two rules is shown in Fig. 1.) Strings match when they either match perfectly (the sequence of 0s and 1s is the same) or when they match partially and the symbols at the indices of the non-matching symbols are the # ‘don’t care’ symbols (see the example in Fig. 1). It should be noted that, whilst the bit string representation is simple, it is nonetheless a perfect representation of the state of an RBN. A bit string representation will not be suitable for all networks however. Fortunately, integer-valued (Wilson 2000b) and real-valued (Wilson 2000a) versions of XCS also exist for networks where nodes are not Boolean-valued.

The subset of population rules that match the current input (RBN state) is termed the ‘match set’. The rules within the match set will suggest a number of actions. The fitness-weighted payoff of each action is used to select a single action to implement in the environment. The match set rules that suggest this single selected action form a subset of the match set called the ‘action set’. The action selected is then fed to an ‘effector’ that performs the desired actions within the environment. Information is then gathered on the payoff associated with the actions taken and the fitness of the rules within the rule collection is updated.

One final mechanism of note must be highlighted. There is a genetic algorithm that interacts with the collection of rules, generating new rules via crossover and mutation, whilst a deletion mechanism prunes the rule set if it gets too large. For more details on Learning Classifier Systems please refer to the work of Urbanowicz et al. (Urbanowicz and Moore 2009). For details on the LCS variant named ‘XCS’ please refer to the work of Wilson (1995, 1998). More details on Genetic Algorithms and Evolutionary Algorithms are available via the work of (Mitchell 1998) and (De Jong 2006).

## Using LCS rule sets to control RBNs

**Fig. 2:**
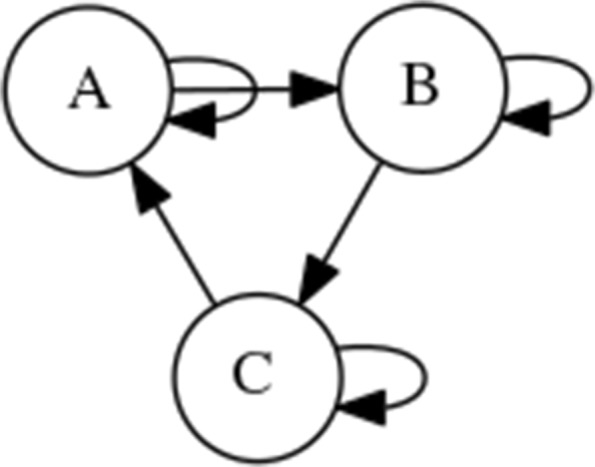
A simple Boolean network

The concept of network motifs has been studied within network science (Gates and Rocha 2016). Both the structural and dynamical properties of these motifs have been examined. Here we briefly use a single network motif – Figure 2D of Gates et al. (Gates and Rocha 2016) – as an illustrative example of a very simple Boolean network in the context of understanding how the system can be controlled. The motif, shown within Fig. 2, simply contains three nodes each with a directed link to the next in a clockwise pattern. In addition to these links, each node also has a directed link to itself.

**Fig. 3:**
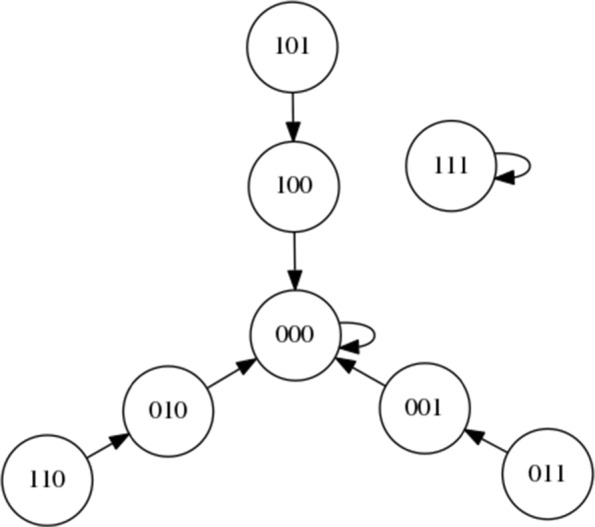
State space of the simple Boolean network with all nodes using the AND logic function

As mentioned in “Random Boolean networks” Section, each node has a value of either 0 or 1. The values of the nodes A, B and C, can be shown as a string indicating the overall status of the network. For instance, if A is set to 0 and B and C set to 1 the status of the network could be shown as 011. When the network is set to an initial string and left to run over a number of steps we can see a trajectory through the system as indicated by a sequence of system states. For instance, in Fig. 3, if the network is initialised at 101 we would see the sequence 101 →100 →000 →000 (the state 000 then continues to repeat). If we re-run the network dynamics multiple times, starting from each single state, we can ‘map out’ the state space, producing a state space graph that clearly shows the relevant attractors. For the simple Boolean network shown in Fig. 2, the resulting state space (when all nodes use the AND function) is shown in Fig. 3. In the example here we can clearly see the attractors as 000 and 111.

**Fig. 4:**
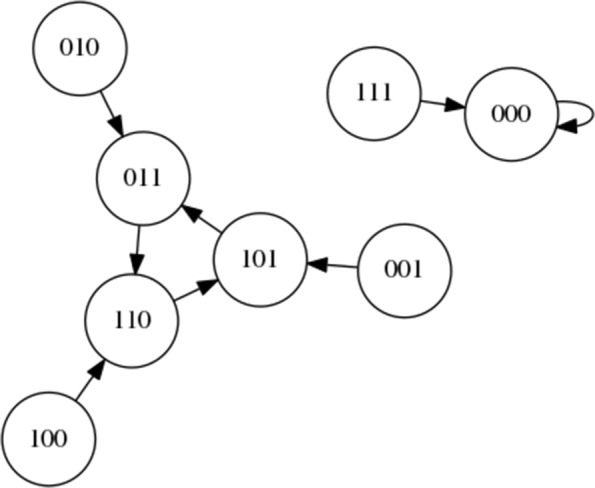
State space of the simple Boolean network with all nodes using the XOR logic function

It should be noted that in Boolean networks all nodes possess a function taking two inputs and outputting a single value. In a *random* Boolean network these functions are random. In Fig. 3 we select only AND functions (for illustration). The AND function outputs a 1 if and only if each input is also a 1 or else outputs a 0. The resulting state space *depends on the precise function at each node*. If XOR (eXclusive OR) functions are used for the network in Fig. 2 then the resulting state space is very different, as shown later in Fig. 4.

Figure 4 presents the state space graph for the simple Boolean network when the XOR function is used for all nodes, instead of the AND function. XOR (exclusive OR) returns a 1 when either input value is a 1. If both input values are 1 or both input values are 0 the function returns a 0. As we can see, the behaviour is more interesting, with a state cycle attractor including the nodes 011, 110, and 101 and a single node attractor at 000.

It is possible to apply the idea of using the Learning Classifier System ternary rules described within “Learning classifier systems” Section to explore the control of a Boolean network. If we again consider the state space within Fig. 4 we can see that if we wish to shift from the single point attractor to the state cycle attractor we need to apply one of three rules: ###:1**;**###:2 or ###:3 (where # represents ‘don’t care’, and the action represents the index of the bit that we wish to flip). In contrast, to reach the single point attractor from the state cycle attractor we can apply one of a number of rules: 110:3**;**011:1**;**101:2**;**001:3**;**010:2 or 100:1. It is worth noting that the rules 110:3**;**011:1 and 101:2 all require that a step be implemented after the intervention, whilst the other rules in the list bring the system to precisely the correct state.

From the above analysis we can see that the number of required control nodes is one, no matter the desired attractor. The example network is very controllable. The current basin of attraction can be altered by just a single bit flip from any location. This is not the case for more complex Boolean networks. Depending on the nodes that are in fact controllable, the controller may have to wait for a number of time steps before the system can be altered (unless more than one control node is available simultaneously, possibly at greater cost).

## Constructing XCS

The above analysis is intended as a demonstration of the application of LCS rules to a Boolean network. However, the rules are intended to work within a full LCS system (specifically XCS herein). In this section we explain the construction of the XCS implementation used in the remainder of the paper. Regrettably, due to space constraints a full description is omitted. Here we aim to describe the critical details of the system, whilst the remaining implementation is covered by the excellent algorithmic description provided by Butz and Wilson (2001).

**Fig. 5:**
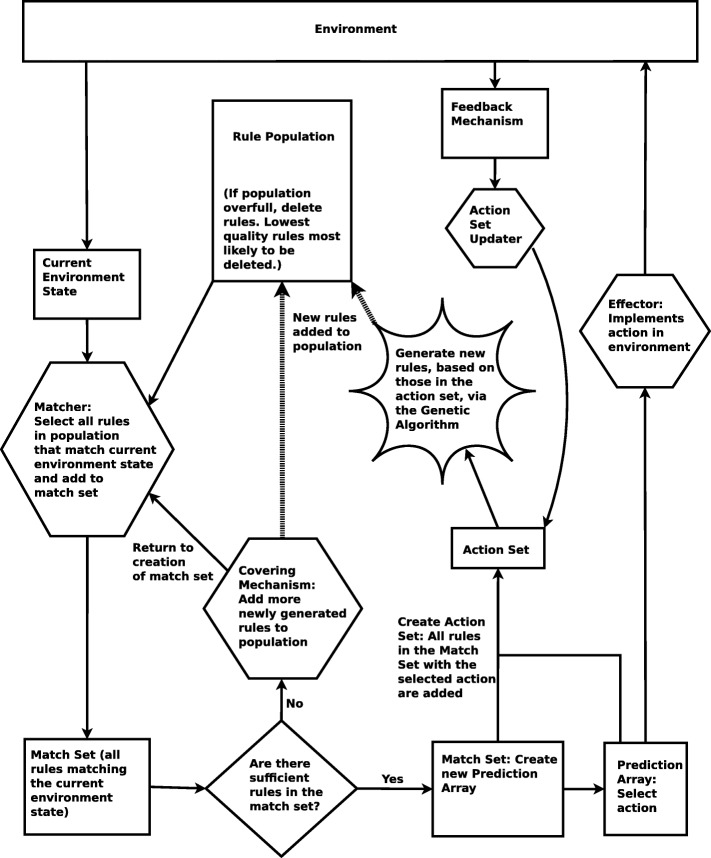
Structure of an XCS classifier system. Based on (Wilson 1998) and originally shown in (Matthew R Karlsen and Sotiris Moschoyiannis: Learning condition action rules for personalised journey recommendations, forthcoming)

A number of components must be outlined in order to form a complete LCS configuration (see Fig. 5). Precise delineation of components varies. Here we base our description on the Butz and Wilson XCS variant that our implementation is built on (Butz and Wilson 2001). Components include a population of ‘classifiers’ (simple state–action rules), a ‘match set’, a ‘prediction array’, an action selection mechanism, an ‘action set’, a rule variable updater, a genetic algorithm, an effector, a ‘covering’ mechanism and a ‘reinforcement program’. These terms are explained throughout the remainder of this section.

The overall mechanics of the program are as follows. For a given network instantiation, a number of trials of the network are run, with the following steps repeated until the target attractor is reached:

- Get the situation from the environment
- Create a match set for the situation
- Create the prediction array from the match set
- Select an action using the prediction array
- Execute the action
- Obtain the payoff value from the environment
- Create the action set for the selected action
- If not first step:
  - Calculate *P* from:
    - the payoff *of the previous step*
    - the max payoff of the current prediction array
  - For each classifier in the *previous* action set, use *P* to:
    - update the classifier’s predicted payoff
    - update the classifier’s error
    - update the classifier’s action set size
  - Use *θ*_*ga*_ to determine whether GA should run on previous action set
  - If GA is to be run, run with the following inputs:
    - the previous action set
    - the population in which to insert new rules
    - the situation *of the previous step*

When the specified attractor is reached, a final update of the action set and a final GA iteration are performed on the *current* action set using the *current* situation and the *current* payoff.

### Detector

The detector simply reads in the current state of the nodes (0 or 1) in an ordered manner such that this order does not change between time steps. The state of the network thus becomes a bit string, such as 0001001010 (for a network of 10 nodes, randomly initialised).

### Rule population

A population of up to *R* rules exists. The rule format combines the bit string provided in the previous section with an index of a node to bit-flip. So, for instance, we may have the rule 1010110101 : 8 which simply means that if the current state of the system is equal to the string on the left, flip bit 8. In this case the bit 8 is a 1 and would be flipped to a 0 by this action. The rules thus look similar to the set shown in Table 1. In XCS, each rule is also associated with five other variables including prediction, error, numerosity, fitness, experience, and an ‘action set size estimate’ (Wilson 1998).

**Table 1:** A population of LCS classifier rules in state-action format

1000101101000111010000001 : 18
1010111001100101001000010 : 11
10111#0011110000011#11101 : 25
0000010110100001101010101 : 14
0101110101010110111110001 : 05
0101110100#01010110110110 : 22
1111##000111110#010101000 : 22
0101100101100010100011000 : 03
0100110110000101010111100 : 09
00011110000##001000110000 : 23

The # symbol indicates ‘don’t care’. In the present context, the left hand condition matches the state of one or more Boolean networks whilst the action integer on the right represents the index of a bit-flip operation to be performed on the Boolean network

### Match set

When an input is fed in to the LCS a match set is generated. This match set is a list of the rules from the population that match the input string. This is either via a direct match, such as when 0101110101 : 5 directly matches the input 0101110101, or an indirect match, such as when 1111##0001 : 7 matches the input 1111000001.

### Prediction array

Once XCS has generated the match set, a fitness-weighted predicted payoff for each action is calculated and stored within the prediction array.

### Action selection

One action must be selected from those in the match set. A number of possible approaches are available for action selection.

Roulette wheel selection is one possibility: buckets proportional to the relative predicted payoffs of each action are created, a bucket is then selected via the drawing of a random number in the range [0,1), the action matching the bucket is then returned.

Another approach is mentioned by Butz and Wilson (2001), whereby with probability *p*_*explr*_ the system explores, selecting a random action from the action set. With probability 1−*p*_*explr*_ the system instead exploits (or functions in a ‘greedy’ manner), selecting the best action, as indicated by the prediction array.

Here we use a deterministic method of selecting whether to perform the best action or a random action. If the predicted payoff is greater than 500, we chose the best action (exploit), whilst if the predicted payoff is less than 500 we choose a random action (explore). In our parameter explorations it was found that this tended to out-perform the probabilistic approach on the problem at hand.

### Action set

Once the action is selected, all rules in the match set with the selected action are forwarded to the action set. If *doActionSetSubsumption* is set to true, action set subsumption occurs, whereby more general classifiers ‘subsume’ less general classifiers. When subsumption occurs the less general classifier is deleted and the more general classifier has its numerosity (instance count) incremented by one. See (Butz and Wilson 2001) for a description of subsumption in the action set. See “Selection of XCS parameters” Section for an explanation of the relevant parameters, *ε*_0_, *θ*_*sub*_, and *β*.

### Rule variable updates

Rule variables (prediction, error, numerosity, fitness, experience, and ‘action set size estimate’) are updated according to the Butz and Wilson pseudocode (Butz and Wilson 2001, p264). *α* and *ν* influence the extent to which prediction error affects fitness (see “Selection of XCS parameters” Section).

### Genetic algorithm

Unlike in other LCSs, the genetic algorithm within XCS is run on the action set rather than the overall population of rules (though the generated rules are still added to the population). If, for this particular action set, the GA has not run for a specified time interval, the GA is activated. The GA functions as follows.

Two ‘parent’ classifiers are selected using roulette wheel selection (described above). ‘Child’ copies are made of these two classifiers. Crossover occurs with probability *χ*, whereby two new rules replace the copies, each formed as a re-combination of the two copies. We then mutate each index position of the new rules with probability *μ*.

If enabled by setting *doGaSubsumption* to true, a ‘subsumption’ check is performed on the two new strings: if either of the parents of each of the new rules is more general than the new rule, with the same action, then the parent’s numerosity is incremented by one and the new rule is not added to the population. When the two new rules are created their prediction, error and fitness values are the average of the two parents values. If one or both new strings are added to the population (if a matching rule already exists in the population the new rule is not added but instead the numerosity of the matching rule in the population is incremented) the population size is assessed. If the population size is found to exceed the maximum, roulette wheel selection is used to select which rules survive, based upon a ‘vote’ for each classifier (Butz and Wilson 2001, p268). (The vote is determined by a number of factors including the ‘action set size estimate’ of the classifier, its experience, fitness and ‘numerosity’.) *δ* determines the fitness threshold at which classifiers are treated differently by the population overspill mechanism whilst *θ*_*del*_ is also similarly used (see “Selection of XCS parameters” Section).

### Effector

The effector simply bit-flips the node with the specified index. So for instance, if the current network state is 00110 and the control rule triggered is 00110 : 2 then the bit at index 2 will be flipped from a 0 to a 1, resulting in the new state 01110. The *action index starts from 1, whilst 0 indicates ‘no action’*.

### Covering mechanism

If the number of matching rules for a given input string is less than *θ*_*mna*_, new rules are added to the population via the covering mechanism. A new rule is created with its condition equal to the environment string and a new random action selected that is not present in the match set. With a certain probability *P*_*#*_ each character of the new rule is mutated in to a # character. New rules are generated in this way until the match set contains the required number of rules. The newly generated classifiers have their predicted payoff, prediction error and fitness initialised to *p*_*I*_, *ε*_*I*_ and *F*_*I*_ respectively.

### Reinforcement program

The reinforcement program (RP) determines the reward associated with a particular action and current environment state. In this system, the reward mechanism functions as follows. Upon initialisation the reinforcement program sets a step count to zero and an intervention count to zero. Each time the RP is required to supply a reward calculation (i.e. once per step) the step count is incremented by one. The intervention count is incremented by one if the action is not 0 (which is the representation of ‘take no action’). When one of the desired attractor states is reached the reward given is simply 1000(*steps*−*interventions*)/*steps*. If one of the desired attractor states is not reached, the reward is zero. The intervention and step counts are reset every time the environment is reset after the target attractor is reached.

### Rule post-processing

To compress the rule set Wilson’s approach described within ‘Compact Rulesets from XCSI’ (Wilson 2001) is adopted. The rule set from the learning classifier is ordered according to numerosity. Then a subset of the rules are applied to the control problem (from every state, to the desired attractor). This set is initially the first rule, with the highest numerosity. If this set fails on any instance, the set size is increased by one (thereby introducing the rule that has the next highest numerosity), and the process is repeated. For completeness, all rule sets in this loop are considered. The rule set with the minimum average number of interventions is recorded as the best solution for our current goals. The result is a compact rule set to control the system.

## Selection of XCS parameters

**R** Maximum population size. Traditionally denoted by *N* but *not* herein due to conflicts with the NK model parameter names. Determines the population size at which deletions start occurring whenever a new classifier is added to the population. If set too high, no selective pressure is applied. If set too low, new rules with potential are removed too quickly from the population. Butz and Wilson (2001) emphasise that this parameter “*should be large enough so that, starting from an empty population, covering occurs only at the very beginning of a run*”.

***γ*** Discount factor. Determines the proportion of the payoff from the present step that is used to reward the rules in the previous action set. If set too low, for multi-step problems such as this one, large ‘action chains’ can not emerge. Put another way, only solutions with a very small number of steps could be learned. If set too high, this also degrades performance. Wilson (1995) sets this to 0.71.

***θ***_***mna***_ Minimum number of actions required in a match set. If the number of actions are less than this, new rules are generated via the covering mechanism. Must never be set greater than the total number of available actions. If set lower than the total number of actions, some actions are not explored for a given state, potentially missing valuable rules. In practice, should be set equal to the number of possible actions.

***P***_***#***_ Probability of inserting hash at a given index when covering. After the initialisation of a new rule the condition perfectly matches the environment. Immediately after this, at each index of the condition, the condition component is converted to a # with a probability of *P*_*#*_. If set too low, few rules will have #s and thus the number of rules required will increase. This may then run up against constraints imposed by *R*. If set too high, overly-general classifiers may come to dominate the population. These overly-general classifiers may succeed in many cases and thus maintain a high fitness even though they do not work in all situations, limiting overall performance. Butz and Wilson (2001) suggest a value of around 0.33 (as used in Wilson’s original experiments).

***p***_***I***_ The initial prediction estimate. New rules have their prediction estimate initialised to this value. If set too high, new rules are treated as well-performing when in fact they are untested. Should be set to zero or very close to zero.

***ε***_***I***_ The initial prediction error. New rules have their prediction error initialised to this value. If set too high, new rules are treated as being inaccurate when this may not be the case. Should be set to zero or very close to zero.

***F***_***I***_ The initial fitness of a rule. New rules have their fitness value initialised to this value. If set too high, the value erroneously marks an untested classifier as worth re-combining via the genetic algorithm. Should be set to zero or very close to zero.

***ε***_**0**_ An error threshold. The error of a classifier (*ε*) must be under this value if it is to be able to subsume other classifiers. If set too high, erroneous classifiers may subsume other classifiers resulting in a loss of good quality classifiers and the strengthening of poor quality classifiers. If set too low, subsumption either never occurs or occurs so rarely that many superfluous rules persist.

This parameter also affects fitness value updates. During the rule variable update, classifiers with an error value (*ε*) of less than *ε*_0_ are awarded an accuracy of 1, thus increasing the classifier’s fitness and its chance to be selected by the GA. If *ε*_0_ is too large here, many classifiers are awarded an accuracy of 1 despite substantial variance in error, thus making the GA less focused (inferior rules are more likely to be selected for reproduction). If set too low, then any payoff variance (resulting in an increase in error) is likely to prevent a rule being reproduced (the GA may effectively become too selective or ‘elitist’) even if the rule has a high payoff.

Butz and Wilson (2001) suggest that this parameter be set to 1% of maximum payoff (maximum payoff is usually 1000). However, in some papers with multi-step problems involving many steps this has been set much lower, albeit with a different fitness update mechanism (Butz et al. 2005).

***θ***_***ga***_ Genetic algorithm activation frequency. If set too high, populations of high quality solutions may be disrupted, resulting in a performance that approaches 100% but never reaches 100% due to continued disruptions. If set too low, the pace of ‘innovation’ inside XCS will be very slow, thereby extending the time required for XCS to become effective in controlling the RBN. Butz and Wilson (2001) suggest a value between 25 and 50, but elsewhere much higher values of around 400 have been used (Butz et al. 2005).

***θ***_***del***_ A classifier age threshold for the deletion mechanism. Classifiers older than *θ*_*del*_ steps are treated differently by the deletion mechanism (if unfit, they are more aggressively removed). If set too high, rules that are definitely inferior may linger in the population rather than being aggressively removed. If set too low, newer rules with uncertain performance may be aggressively removed despite this uncertainty. Butz and Wilson (Butz and Wilson 2001) suggest an approximate value of 20.

***β*** The higher this is set, the earlier classifiers are treated with the ‘main’ update rule for updating their properties (fitness, performance, error). The two-tier update rules enable the initial values of fitness, performance and error to reach accurate values more rapidly (p153 of (Wilson 1995)). This means that if *β* is set too high, inaccuracies in the initial values of fitness, performance and error become more important. If set too low, the main update rule will not be used when it should, resulting less accurate updates of fitness, performance and error. Wilson (1995) uses a setting of 0.2. Parameter exploration in this problem, and in the problem of making personalised recommendations to rail passengers for their onward journies (Matthew R Karlsen and Sotiris Moschoyiannis: Learning condition action rules for personalised journey recommendations, forthcoming), suggests that most successful parameter combinations have *β* close to this value.

**Fig. 6:**
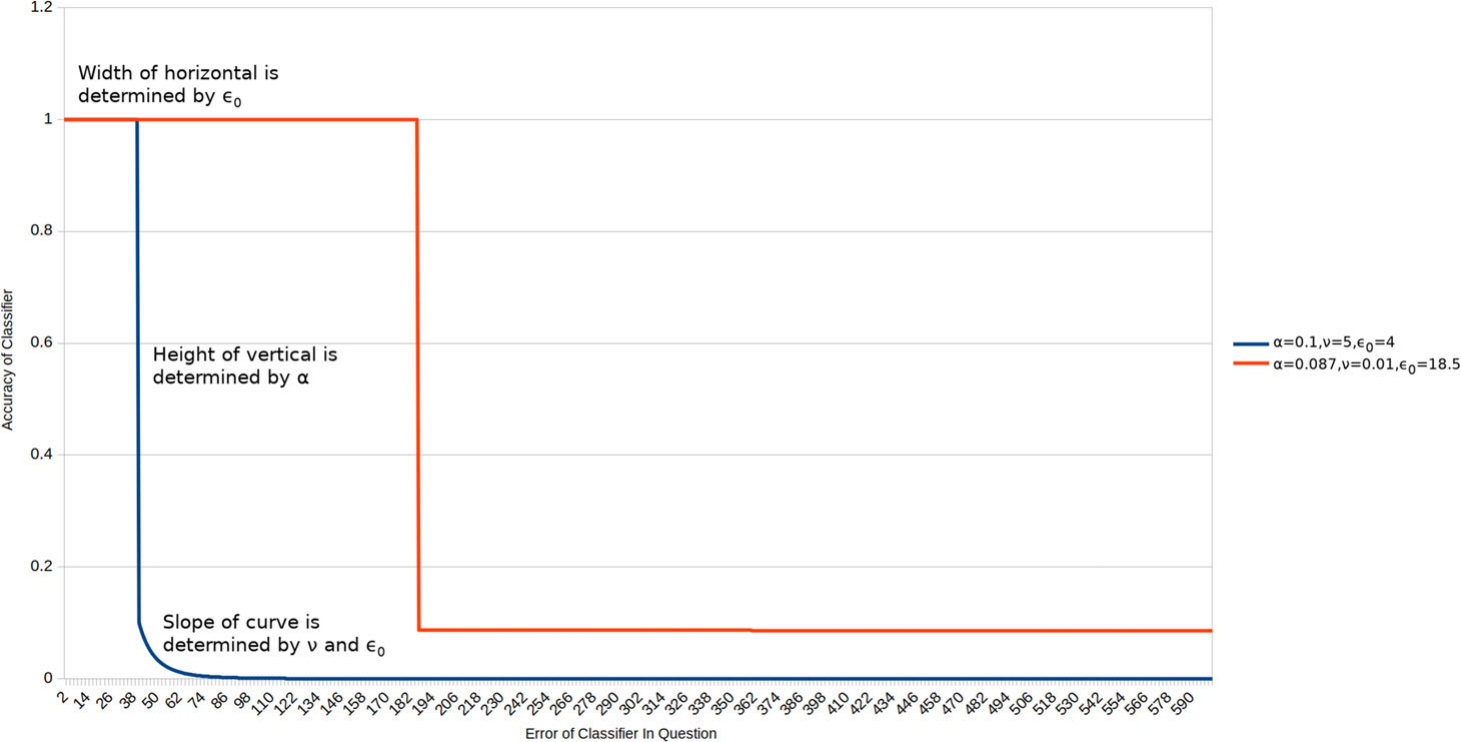
A chart showing the relationship between a classifier’s error and its accuracy for the XCS classifier accuracy function. Accuracy is defined as: *α*(*ε*_*j*_/*ε*_0_)^−*ν*^ where *ε*_*j*_ is the error of classifier j. This chart is based on that presented by Butz et al. Fig. 1 (Butz et al. 2001)

***α*** Affects the calculation of the classifier’s fitness as follows. A classifier’s accuracy is crucial in determining its fitness. Accuracy depends on the classifier’s error, the value of *ε*_0_, *α*, and *ν*. When a classifier’s error is below *ε*_0_ accuracy is set to 1. When a classifier’s error is greater than or equal to *ε*_0_, the accuracy is determined by the settings of *α* and *ν*. If *α* is high then there will be a large ‘drop off’ when a classifier’s error exceeds one. In contrast, when *α* is high (approaching 1), there is very little drop off and the rate of accuracy decrease as error increases is determined almost completely by *ν*. See Fig. 6 for an intuitive representation.

***ν*** Affects fitness value updates. Butz and Wilson (2000) advise a value of 5. Determines how quickly accuracy falls to zero after the drop off (where the classifier’s error exceeds *ε*_0_). If high, accuracy falls rapidly. If low, accuracy falls slowly. See Fig. 6 for an intuitive representation.

***χ*** The likelihood of crossover being applied when the GA is run. If set high, crossover will always occur. This may be desirable. If set too low, rules are never crossed over or are crossed over infrequently, reducing the likelihood of fitter, novel, recombinations of existing rule conditions. Assuming that the problem is of the type such that recombination of good solutions can produce even better solutions, a low crossover rate could decrease the speed at which the rule population is improved. In practice the authors would suggest 0.70–0.90 for this parameter, in line with the suggested range of 0.5–1.0 by Butz and Wilson (2000).

***μ*** Likelihood of mutation at each index of a solution newly-created by the GA. If set too high, new solutions essentially become unrelated to the good solutions they are based off – the GA becomes almost equivalent to the covering mechanism rather than adjusting good solutions. If set too low, the new solutions based off good solutions are almost always facsimiles of their parents, and thus the GA does not generate novelty or explore new areas of the rule space. Wilson (1995) uses the value 0.01 (for the Woods2 multi-step problem).

***δ*** Determines the fitness ‘cut off point’ below which rules are more aggressively deleted (p154 of (Wilson 1995)). If set too high, poor quality rules may be preserved unnecessarily. If set too low, rules with potential may be deleted before they are adequately evaluated. Wilson (1995) uses the value 0.1.

***θ***_***sub***_ Subsumption-related parameter. Determines how long classifiers must exist in the population before they can subsume other classifiers. If set too high, subsumption takes too long and thus superfluous classifiers persist. If set too low, overly-general classifiers may out compete less-general but higher performing classifiers. Butz and Wilson (2001) suggest an approximate value of 20. In practice, for longer multi-step problems, a value higher than this may be better.

***p***_***explr***_ Probability of exploration. Determines whether a random action is selected rather than the predicted best action. If set too low, XCS potentially latches on to sub-optimal solutions without exploring a wider range of actions. If set too high, performance suffers because the behaviour of the system essentially becomes random. In practice, an exploration rate of 0.5, or slightly lower, seems to be most widely used. This variable *is a parameter of the original XCS* – here we use a slightly different approach (as explained earlier).

***doActionSetSubsumption*** Perform subsumption in the action sets. If set to true, less general rules are subsumed by more general rules in the action set (i.e. the less general rule is deleted and the numerosity of the more general rule is incremented by one). Subsumption is beneficial because it compresses the rule set, removing superfluous rules. Subsumption can be detrimental because it can enable *overly* general rules to take over the population, eliminating new promising rules before they become established. Some authors, such as Lanzi (1999), have found performance to be higher with action set subsumption disabled.

***doGaSubsumption*** Perform subsumption in the GA. The advantages and disadvantages are similar to those of action set subsumption. Since the GA is applied far less frequently than the frequency of action set generation, GA subsumption tends to be less problematic than action set subsumption.

## Experiments

In this section we describe our experiments in applying the proposed XCS learning classifier systems to a number of Boolean network instances. These provide insight into the evolution of the rule set, and resulting interventions, that takes the network from any state to a desired state (attractor) or state cycle. The experiments where run on a laptop with an i7–7700HQ (2.80 gigahertz) processor with 16 gigabytes of random access memory. The program itself was written in the Java programming language.

### Parameter settings

The parameter settings described here apply to the XCS variant used for the experiments. The initial parameter settings used were aquired from the Butz and Wilson paper (Butz and Wilson 2001). Final parameter settings where acquired via the use of a random parameter space explorer. Through substantial manual exploration we have been able to find parameter settings of comparable performance but none that are definitively better. Final parameters are thus set as in Table 2.

**Table 2:** Parameter settings and brief descriptions

Parameter	Value	Brief Description
*R*	790	Rule population size
*γ*	0.76	Discount rate
*θ*_*mna*_	6	Min. number of actions in match set
*P*_*#*_	0.4	Probability of hash
*p*_*I*_	7.4	Initial payoff
*ε*_*I*_	1.0	Initial error
*F*_*I*_	0.03	Initial fitness
*ε*_0_	18.5	Error threshold
*θ*_*ga*_	260	Genetic algorithm frequency
*θ*_*del*_	32	Deletion threshold
*β*	0.01	Affects update of *p*, *ε*, and action set size for classifiers
*α*	0.087	Affects fitness updates
*ν*	0.01	Affects fitness updates
*χ*	0.711	Likelihood of GA crossover operation
*μ*	0.263	Likelihood of GA mutation operation
*δ*	0.05	Modifies the effect of fitness on the ‘deletion vote’ of a classifier
*θ*_*sub*_	31.579	Subsumption threshold
*p*_*explr*_	N/A	Likelihood of exploring
*doActionSetSubsumption*	true	Perform subsumption in the action set?
*doGaSubsumption*	true	Perform subsumption in the GA?
*useNewActionChooser*	true	Use the new action chooser mechanism?

Here we must make a note on the unusual combination of *α* (0.087), *ν* (0.01) and *ε*_0_ (18.5) and explain why we believe this combination works well on the present problem. As explained previously, our payoff function in the ‘reinforcement program’ is 1000(*steps*−*interventions*)/*steps*. This means that for a given rule, payoff may fluctuate (sometimes the rule is used in an ‘action chain’ that involves many steps and sometimes it is used in a chain that involves just a few steps). This tends to increase the error of the classifier in question. In order to preserve these classifiers, the above parameter combination is more tolerant of error than is usual. Future work should involve an approach that adopts a payoff function that avoids these fluctuations *whilst at the same time* devising a method to minimise the number of active interventions (which is what the present payoff function is designed to do).

### Network structures

**Fig. 7:**
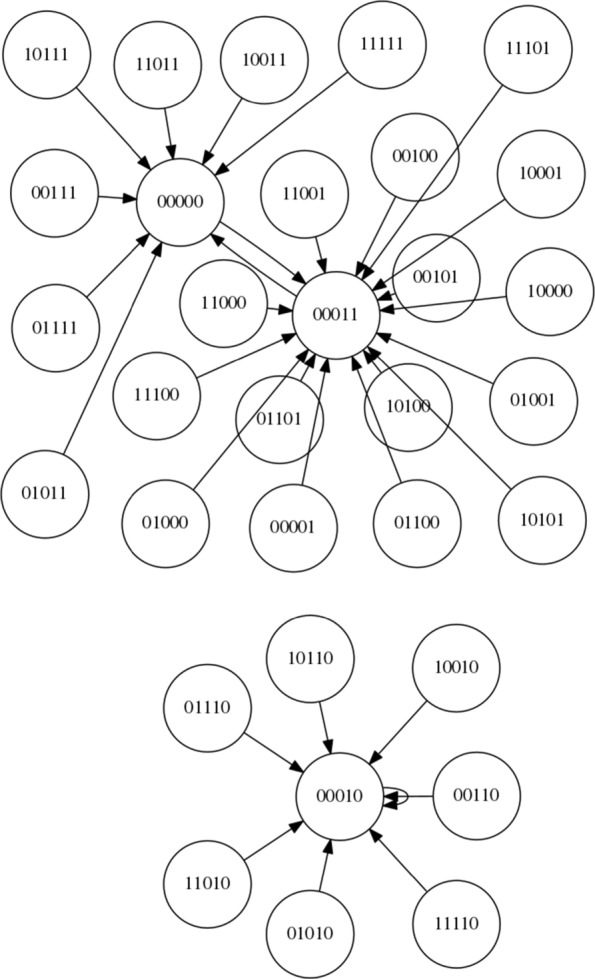
NK Boolean network, instantiation 1 (*N*=5,*K*=2)

**Fig. 8:**
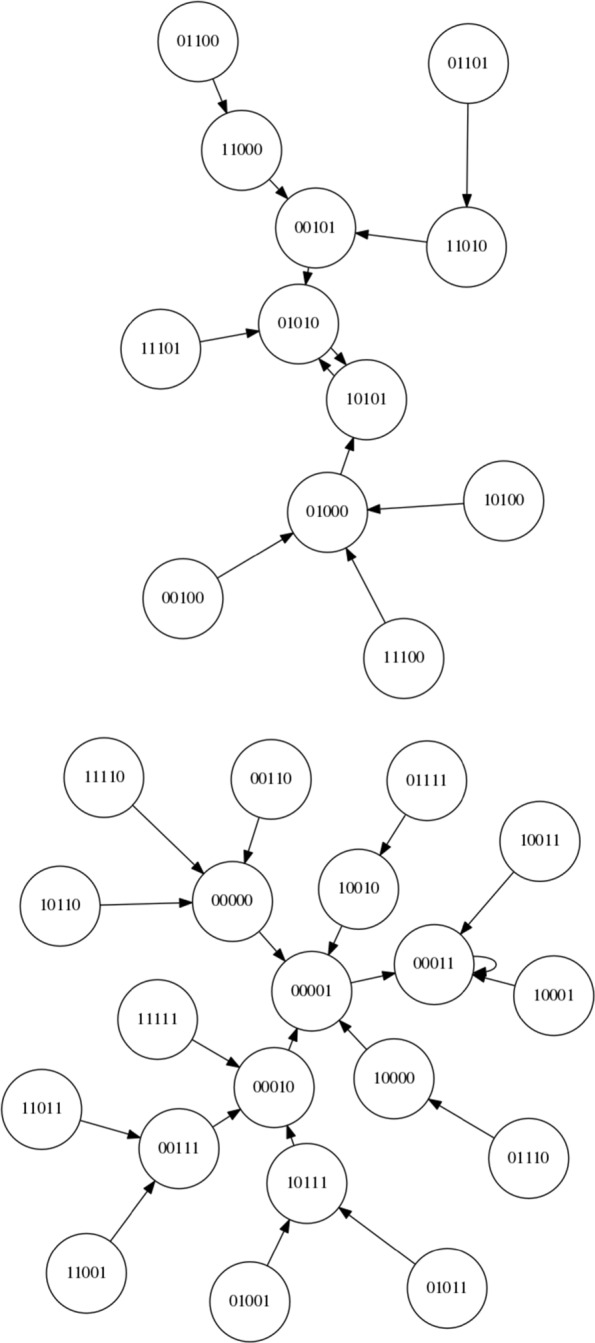
NK Boolean network, instantiation 2 (*N*=5,*K*=2)

**Fig. 9:**
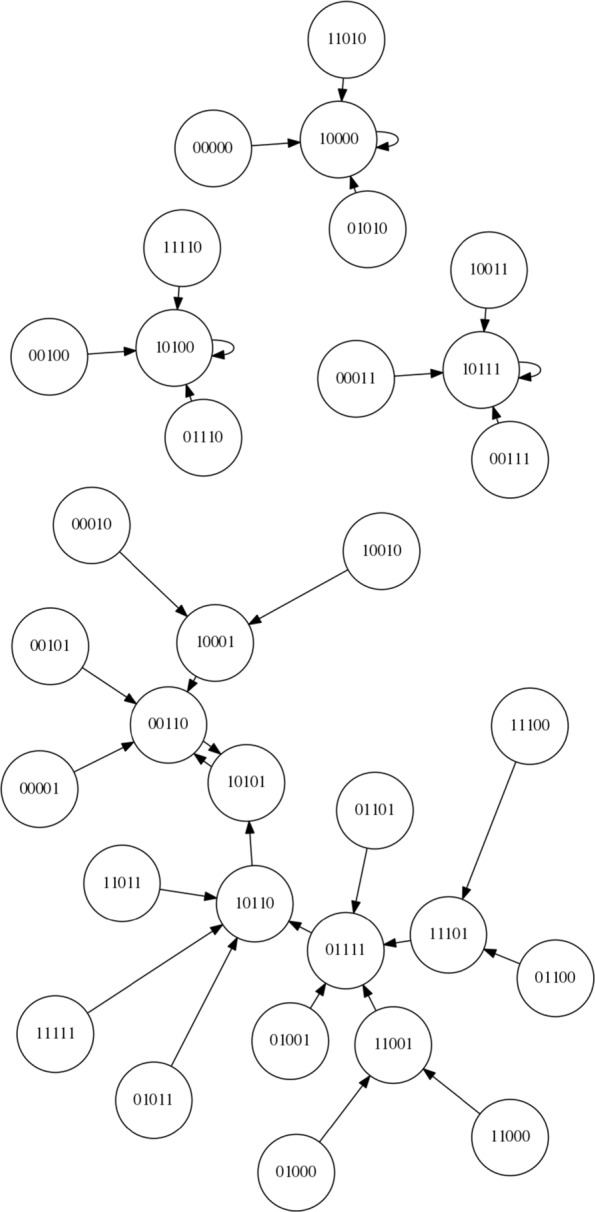
NK Boolean network, instantiation 3 (*N*=5,*K*=2)

**Fig. 10:**
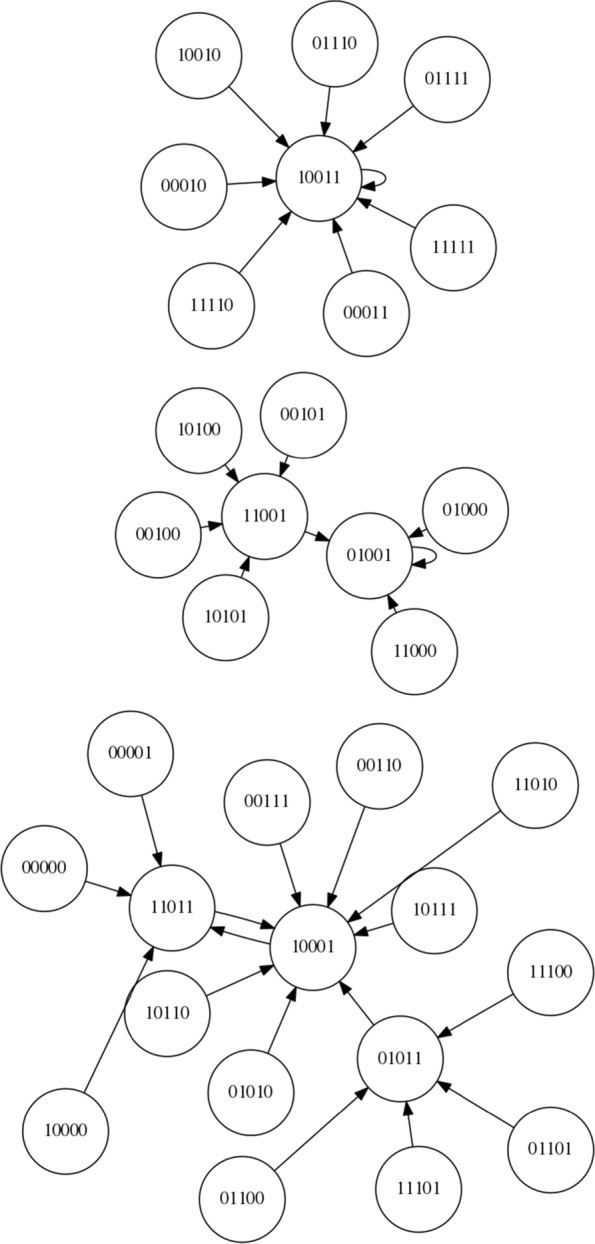
NK Boolean network, instantiation 4 (*N*=5,*K*=2)

Since this is, to our knowledge, the first time an LCS has been applied to controlling an RBN here we focus on a simple boolean network with *N* = 5 and *K* = 2 and synchronous updating. The network is instantiated according to the description within “Random Boolean networks” Section. Using these settings, 4 network configurations are created and stored for re-use and future reference. The state spaces of these networks are shown in Figs. 7, 8, 9 and 10. For each of the networks a target attractor (or state cycle) is selected. The LCS and post-processing is run 5 times on each network, for at least 250,000 trials on each run, and a summary of the resulting control rules and associated statistics are presented in the next section. Each ‘trial’ of the process involves instantiating the RBN at a random initial state and then performing action steps or ‘natural’ steps until the desired attractor is reached. When an action step is performed a ‘natural’ step is immediately performed after the action (it is not possible to perform multiple action steps at once within the current framework, by design rather than technical limitation).

Due to a limitation of the XCS system there is no guarantee that XCS currently evolves rules that completely cover the state space. For this reason it is sometimes necessary to run XCS for greater than 250,000 steps to acquire a solution. With good parameter settings this is a very rare event. Our XCS implementation is designed to evaluate the rule set after 250,000 steps and then every 10,000 steps thereafter. If the solution covers the state space and produces no failures then the system moves on to the post-processing stage. Future work could improve this behaviour.

Figure 7 presents the state space of the first network instantiation. An inspection of the graph structure reveals two attractors, 00011 →00000 →00011 and 00010 →00010. As the target attractor for this state graph, we select the attractor with the smallest basin of attraction, 00010 →00010, representing the greatest challenge. This network is comparatively simple compared to some other networks, with an opportunity to migrate from the larger to the smaller basin at both 00000 and 00011. Perhaps the greater challenge on this simple network is to avoid making unnecessary interventions when they are not required.

Figure 8 presents the state space of the second network instantiation. An inspection of the graph structure again reveals two attractors, 00011 →00011 and 01010 →10101 →01010. The target attractor is selected as 01010 →10101 →01010. We define the bit-flip distance between two attractors as the minimum number of simultaneous bit flip actions that would be required to modify any one state in one attractor to any one state in the other attractor directly. The bit-flip distance between the attractors is 2 (i.e. two simultaneous bit flip actions) and therefore the LCS cannot ‘travel’ from one attractor to the other by a single intervention in the system – other routes must be found.

Figure 9 presents the state space of the third network instantiation. An inspection of the graph reveals four attractors, 00110 →10101 →00110, 10111 →10111, 10100 →10100, and 10000 →10000. We select the target attractor as 10111, which has a bit-flip distance of 1 from the 00110 →10101 →00110 attractor, a bit-flip distance of 2 from the 10100 →10100 attractor and a distance of 3 from the 10000 →10000 attractor.

Figure 10 presents the state space of the fourth network instantiation. An inspection of the graph reveals three attractors, 11011 →10001 →11011, 10011 →10011 and 01001 →01001. We select the attractor 01001 →01001 as the target attractor, which has a bit-flip distance of 2 from 11011 →10001 →11011 and a bit-flip distance of 3 from 10011 →10011.

## Results and Discussion

### Resulting control rules

Table 3 presents the results of the 20 LCS runs (5 runs of 250,000 or more trials on 4 different networks). The table provides statistics for the rule set that minimises the number of interventions in the system. The column headings are as follows: **Net** indicates the network instance number, **Run** indicates the run number, **CR** indicates the compressed number of rules required to *minimise the number of average interventions* (from those rule combinations evaluated), **AS** indicates the average number of steps required to reach the attractor with this rule set, **AI** is the average number of interventions required to reach the attractor with this rule set, and **Time** is the complete time taken to produce the rule set in seconds.

**Table 3:** Performance statistics for the four different network structures, with five runs per structure

Net	Run	CR	AS	AI	Time (s)
1	1	10	2.938	1.0	23
1	2	17	2.844	1.208	22
1	3	11	2.938	1.333	23
1	4	12	2.781	1.167	24
1	5	12	2.938	1.0	22
2	1	16	4.438	1.1	113
2	2	304	4.531	1.333	120
2	3	15	4.531	1.15	117
2	4	16	4.438	1.1	117
2	5	20	4.5	1.182	115
3	1	35	3.719	1.286	55
3	2	14	3.938	1.286	54
3	3	25	3.844	1.286	54
3	4	14	4.438	1.572	57
3	5	16	3.813	1.286	57
4	1	18	4.313	1.714	64
4	2	18	3.938	1.714	67
4	3	168	3.594	1.5	65
4	4	20	3.938	1.714	63
4	5	13	4.313	1.714	65

The step count includes any active interventions (the application of one of the actions in the range 1 to 5) and the ‘natural’ steps that occur during the evolution of the system. The action ‘0’ (i.e. no action) is not counted as a step or intervention. *Each control step is always followed by at least one ‘natural’ step.* This emerges as a result of our restriction requiring that only a single intervention is permitted at once (permitting multiple interventions would reduce the difficulty of the problem).

**Fig. 11:**
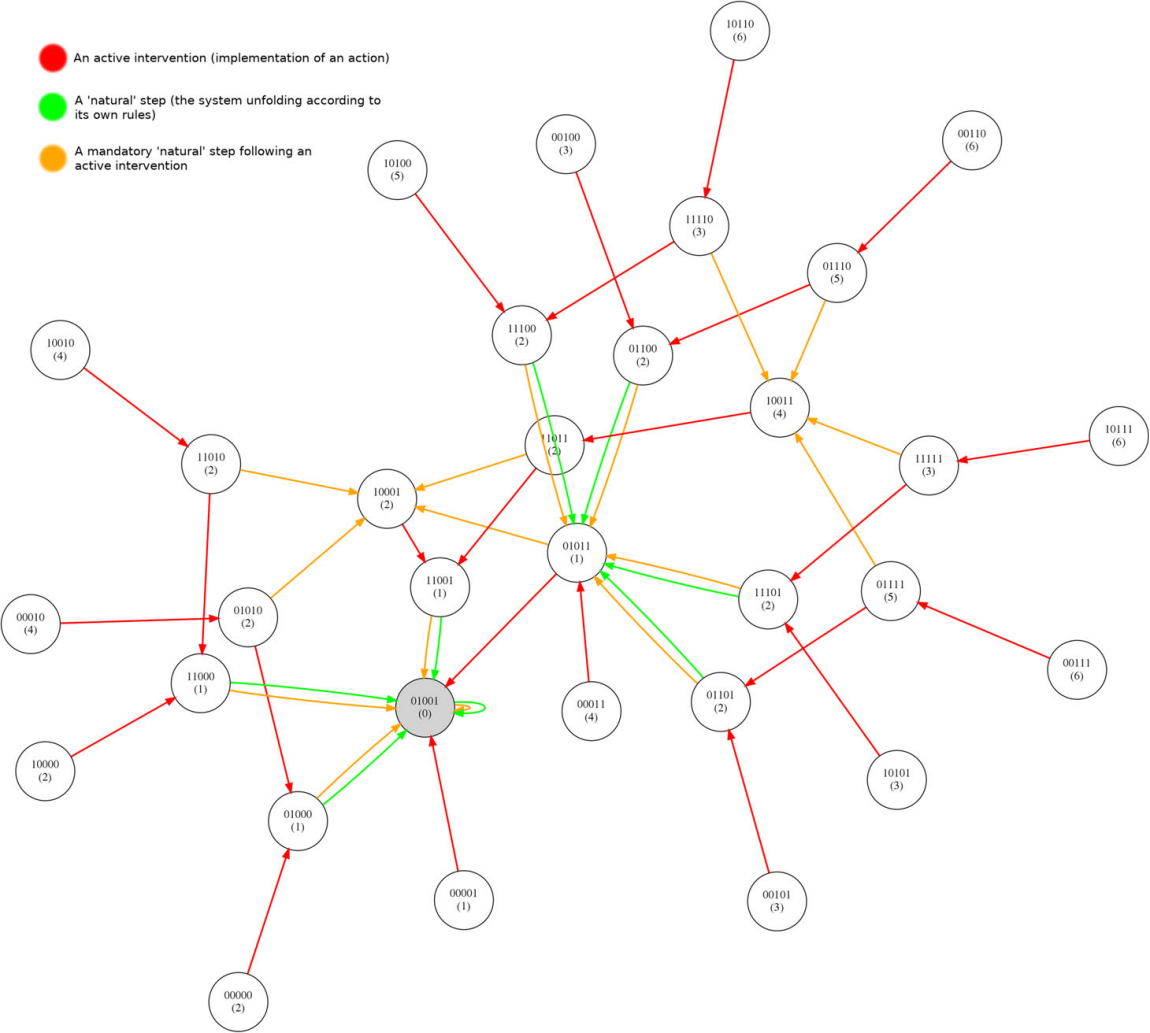
An example control graph for NK Boolean Network 4 using an evolved rule set

We now present a number of the specific rule sets that have been evolved. For network instance 1 rule set presented in Table 4 was evolved. Table 5 presents the rule sets evolved for instance 2. For instance 3 the rule sets presented in Table 6 were evolved. For instance 4 the rule sets presented in Table 7 were evolved. Finally, in Fig. 11 we present an application of one randomly evolved rule set for network 4 to the network graph. The rule set is able to guide the system from any state to the specified attractor.

**Table 4:** Rule sets evolved for the 1st network instance, shown with [performance estimate, fitness]

Set 1	Set 2	Set 3	Set 4	Set 5
##### : 1 [94.67/0.9031]	##### : 3 [106.62/0.9998]	##### : 2 [101.2/0.9999]	##### : 2 [102.59/0.9999]	##### : 1 [106.46/0.9691]
##### : 2 [96.39/0.999]	##### : 1 [100.4/1]	##### : 1 [104.67/0.9999]	##### : 3 [101.54/1]	##### : 3 [106.86/0.9907]
##### : 3 [95.01/0.9968]	##### : 2 [97.51/1]	##### : 3 [101.57/1]	##### : 1 [98.45/0.9994]	##### : 2 [105.79/0.9885]
####0 : 5 [103.31/0.8209]	###0# : 0 [100.08/0.8696]	####1 : 0 [98.06/0.8865]	####1 : 0 [104.46/0.9143]	####1 : 0 [110.8/0.9087]
####1 : 0 [101/0.8834]	###1# : 4 [99.61/0.6823]	###0# : 0 [104.69/0.878]	###0# : 0 [98.97/0.9045]	###0# : 0 [99.03/0.9037]
###0# : 0 [98.82/0.8742]	####1 : 0 [104.48/0.8532]	####0 : 5 [106.82/0.6445]	###1# : 4 [101.47/0.6664]	####0 : 5 [107.1/0.7033]
###1# : 4 [100.56/0.5989]	####0 : 5 [102.1/0.6612]	###1# : 4 [108.2/0.6296]	####0 : 5 [100.98/0.7138]	###1# : 4 [102.56/0.6743]
####1 : 4 [97.87/0.4512]	####1 : 4 [100.21/0.3237]	###0# : 5 [105.84/0.3973]	####1 : 4 [99.05/0.3768]	###0# : 5 [98.76/0.3346]
##### : 0 [135.11/0.0642]	###0# : 5 [100.38/0.3645]	####1 : 4 [104.92/0.409]	##### : 5 [114.67/0.077]	####1 : 4 [98.63/0.3579]
##### : 4 [117.54/0.0547]	##### : 0 [173.14/0.0583]	##### : 0 [178.02/0.0632]	###0# : 5 [99.32/0.2865]	##### : 5 [116.26/0.0925]
	##### : 4 [109.75/0.0622]	##### : 5 [126.9/0.0724]	##### : 0 [158.1/0.0458]	##### : 4 [118.29/0.0599]
	##### : 5 [127.45/0.0594]		##0## : 4 [120.77/0.0474]	##### : 0 [225.99/0.0472]
	##0## : 0 [173.14/0.0399]			
	0#### : 5 [127.45/0.0505]			
	0#### : 0 [173.14/0.0342]			
	##0## : 5 [127.45/0.0375]			
	#0### : 0 [173.14/0.0279]

**Table 5:** Rule sets evolved for the 2nd network instance, shown with [performance estimate, fitness]

Set 1	Set 2	Set 3	Set 4	Set 5
##### : 5 [168.77/1]	##### : 5 [167.08/0.9999]	##### : 1 [140.22/0.837]	##### : 5 [176.32/0.9964]	##### : 5 [158.27/1]
####1 : 2 [174.82/0.9105]	####1 : 2 [165.75/0.9181]	##### : 5 [165.66/0.9237]	####1 : 2 [166.58/0.8826]	####1 : 2 [164.28/0.9272]
####0 : 1 [174.58/0.7241]	####0 : 1 [179.69/0.8753]	####1 : 2 [152.97/0.9133]	####0 : 1 [179.2/0.8245]	###1# : 3 [154.08/0.8235]
###1# : 3 [161.39/0.7999]	###1# : 3 [159.32/0.8038]	###1# : 3 [152/0.7947]	###1# : 3 [157.67/0.8161]	####0 : 1 [172.67/0.7602]
####1 : 1 [150.51/0.3144]	####1 : 1 [134.84/0.5842]	#1### : 2 [174.74/0.8665]	####1 : 1 [145.04/0.349]	####1 : 1 [138.2/0.3596]
#1### : 2 [171.8/0.8389]	#1### : 2 [175.93/0.8522]	##1## : 3 [130.54/0.5219]	#1### : 2 [172.82/0.8533]	#1### : 2 [169.65/0.8835]
##1## : 3 [138.72/0.5171]	##1## : 3 [135.56/0.5524]	#00## : 4 [136.33/0.3224]	##1## : 3 [134.61/0.5635]	##1## : 3 [134.61/0.5405]
###1# : 1 [151.82/0.3025]	####0 : 3 [180.32/0.0755]	####0 : 3 [173.76/0.0651]	##### : 2 [185.62/0.0787]	#00## : 4 [136.53/0.4471]
##0## : 1 [148.58/0.1909]	#1### : 3 [171.64/0.3477]	#00## : 0 [137.08/0.3773]	###1# : 1 [157.57/0.1491]	#1### : 3 [163.03/0.3735]
#00## : 0 [146.3/0.4662]	#00## : 4 [138.48/0.3762]	#1### : 3 [166.97/0.3807]	##### : 1 [159.29/0.1256]	##0## : 1 [141.51/0.1193]
#1### : 3 [178.45/0.3704]	#00## : 0 [140.19/0.4581]	##### : 2 [174.49/0.055]	##0## : 1 [153.58/0.12]	##### : 1 [145.76/0.1253]
##### : 3 [187.29/0.0621]	###11 : 0 [138.38/0.3636]	##### : 4 [164.29/0.0881]	#00## : 4 [144.24/0.489]	###1# : 1 [148.31/0.1103]
##### : 2 [183.11/0.0487]	###1# : 2 [180.27/0.0468]	##### : 0 [187.87/0.0619]	##### : 4 [169.22/0.0742]	##### : 0 [177.05/0.0695]
#00## : 4 [143.36/0.3858]	##### : 1 [144.49/0.0455]	1#### : 3 [140.72/0.0686]	#00## : 0 [146.89/0.4448]	#00## : 0 [133.87/0.4369]
##### : 1 [157.12/0.0928]	##### : 2 [179.39/0.0434]	##### : 3 [175.77/0.0436]	##### : 0 [198.09/0.0717]	##### : 4 [154.1/0.0629]
##### : 0 [193.18/0.0486]	##### : 4 [152.35/0.0524]		##### : 3 [195.4/0.0698]	####0 : 3 [173.23/0.0527]
	##0## : 1 [143.81/0.0449]			0#### : 1 [141.69/0.0865]
	##### : 3 [177.73/0.0475]			#0##0 : 0 [168.55/0.343]
	###1# : 1 [147.84/0.0443]			##### : 3 [172.64/0.0496]
	##0## : 0 [158.01/0.0401]			##### : 2 [188.22/0.0433]
	#0### : 1 [141.28/0.0422]			
	###01 : 4 [140.66/0.2853]			
	##### : 0 [162.97/0.0379]			
	#0### : 4 [147.31/0.0256]			
	0#### : 1 [142.66/0.0267]			
	####1 : 3 [181.42/0.0291]			
	⋯

**Table 6:** Rule sets evolved for the 3rd network instance, shown with [performance estimate, fitness]

Set 1	Set 2	Set 3	Set 4	Set 5
##### : 2 [121.82/0.9639]	##### : 1 [101.26/0.9999]	##### : 3 [116/1]	##### : 3 [114.15/0.997]	##### : 2 [125.42/0.9657]
##### : 3 [117.52/0.9997]	##### : 2 [104.75/0.8532]	##### : 2 [112.65/0.974]	##### : 1 [112.65/1]	##### : 1 [117.51/0.9971]
##### : 0 [134.03/0.7818]	##### : 3 [106.64/0.9994]	##### : 1 [111.3/0.9999]	##### : 2 [122.79/0.6266]	##### : 3 [113.6/0.9991]
##### : 1 [115.17/0.9897]	##### : 0 [119.85/0.3689]	##### : 0 [131.67/0.6145]	##### : 0 [133.09/0.3867]	##### : 0 [136.59/0.6155]
####1 : 5 [103.8/0.7621]	####1 : 5 [93.2/0.7667]	####1 : 5 [100.29/0.7831]	####1 : 5 [100.13/0.6399]	####1 : 5 [107.98/0.804]
####0 : 4 [107.45/0.6914]	####0 : 4 [97.61/0.6584]	####0 : 4 [104.67/0.684]	####0 : 4 [104.39/0.7047]	###0# : 5 [125.68/0.2339]
###0# : 5 [120.92/0.2807]	###0# : 5 [111.04/0.2269]	###0# : 5 [119.14/0.2628]	###0# : 5 [116.14/0.5845]	####0 : 4 [112.33/0.6788]
###1# : 4 [108.52/0.4822]	###1# : 4 [97.18/0.5201]	###1# : 4 [105.32/0.5476]	#1### : 4 [112.93/0.6228]	##### : 4 [132.25/0.1083]
#1### : 4 [110.69/0.5677]	#1### : 4 [99.77/0.5808]	#1### : 4 [103.8/0.5249]	###1# : 4 [106.4/0.4999]	#1### : 4 [113.37/0.6087]
##### : 5 [139.27/0.1241]	###0# : 0 [105.38/0.6715]	##1## : 4 [116.63/0.0589]	##### : 4 [131.46/0.0603]	###1# : 4 [111.4/0.4896]
##1## : 4 [123.69/0.0815]	##### : 4 [108.83/0.0727]	##### : 4 [121.25/0.0614]	##### : 5 [136.74/0.0604]	##### : 5 [156.44/0.1187]
##### : 4 [128.74/0.064]	##### : 5 [121.41/0.0921]	##### : 5 [139.96/0.1052]	#1### : 5 [100.04/0.1964]	#1### : 5 [100.35/0.201]
0#### : 4 [102.19/0.165]	##1## : 4 [106.3/0.045]	#1### : 5 [95.69/0.2266]	##1## : 4 [124.77/0.0427]	##1## : 4 [122.66/0.0409]
#1### : 5 [100.08/0.1717]	#1### : 5 [91.22/0.2257]	1#### : 5 [120.22/0.0714]	####0 : 0 [101.49/0.4351]	0#### : 4 [105.27/0.1486]
1#### : 5 [125.82/0.0498]		0#### : 4 [97.35/0.1286]		##1## : 5 [162.55/0.0484]
####0 : 5 [174.41/0.071]		####1 : 4 [137.57/0.0457]		####0 : 5 [189.87/0.0748]
1#### : 4 [134.03/0.0248]		#0### : 5 [146.21/0.0445]		
###1# : 5 [164.51/0.0373]		##1## : 5 [143.43/0.0383]		
##0## : 5 [117.48/0.117]		1#### : 4 [128.34/0.0269]		
#0### : 5 [148.1/0.037]		###1# : 5 [160.52/0.0381]		
#0### : 4 [129.9/0.0204]		#0### : 4 [124.37/0.0238]		
###0# : 4 [138.3/0.0258]		####0 : 5 [173.3/0.0553]		
####1 : 4 [145.61/0.0284]		##0## : 5 [115.58/0.0305]		
##1## : 5 [142.67/0.0307]		###0# : 4 [133.83/0.0302]		
#0##0 : 5 [174.41/0.0478]		###0# : 0 [113.13/0.3142]		
10### : 4 [134.29/0.0214]				
###01 : 4 [169.18/0.0349]				
1##0# : 4 [138.3/0.0187]				
0##1# : 5 [185.65/0.0263]				
0#### : 5 [185.65/0.0244]				
1###1 : 4 [155.32/0.0193]				
##110 : 5 [209.89/0.031]				
##0## : 4 [159.54/0.0158]				
10#01 : 4 [189.52/0.0323]				
1###0 : 5 [145.4/0.0391]

**Table 7:** Rule sets evolved for the 4th network instance, shown with [performance estimate, fitness]

Set 1	Set 2	Set 3	Set 4	Set 5
##### : 3 [158.04/0.6912]	##### : 3 [161.12/0.7789]	##### : 3 [151.87/0.8591]	##### : 3 [164.89/0.9302]	##### : 3 [178.43/0.7687]
#0### : 4 [143.15/0.8093]	#0### : 4 [143.89/0.8566]	#0### : 0 [138.66/0.5465]	#0### : 4 [145.93/0.874]	##### : 5 [166.26/0.4916]
#1### : 2 [141.8/0.6631]	##### : 1 [148.57/0.1628]	#0### : 1 [137.04/0.6962]	#0### : 0 [143.99/0.671]	#0### : 0 [155.22/0.7768]
#0### : 0 [142.02/0.7156]	#0### : 0 [141.8/0.7258]	#0### : 5 [139.48/0.5659]	#0### : 1 [144.31/0.6944]	#0### : 1 [156.72/0.6967]
#0### : 1 [141.68/0.6972]	#1### : 2 [141.97/0.656]	###0# : 4 [133.7/0.7053]	#1### : 2 [146.17/0.5975]	###1# : 2 [161.67/0.3856]
###1# : 0 [147.07/0.6095]	#0### : 5 [142.08/0.7452]	###1# : 2 [137.47/0.7656]	#0### : 5 [144.57/0.6855]	#0### : 4 [157.3/0.845]
###1# : 1 [147.51/0.4956]	###1# : 0 [148.65/0.5244]	#0### : 4 [140.73/0.5582]	###1# : 0 [149.63/0.6199]	###1# : 0 [160.97/0.4031]
###0# : 4 [144.92/0.3457]	###1# : 5 [149.53/0.4156]	#1### : 2 [134.26/0.3745]	###1# : 2 [152.96/0.4794]	###1# : 1 [160.68/0.5334]
###1# : 5 [146.55/0.6291]	#0### : 1 [140.34/0.6444]	###1# : 1 [133.32/0.601]	###1# : 1 [150.01/0.5601]	#1### : 2 [154.82/0.7113]
###1# : 2 [149.37/0.5411]	###0# : 4 [148.5/0.2268]	###1# : 0 [135.38/0.6634]	###0# : 4 [148.19/0.1629]	###0# : 4 [156.76/0.1942]
##### : 5 [150.95/0.1049]	###1# : 2 [150.1/0.4271]	###1# : 5 [136.7/0.6408]	###1# : 5 [149.3/0.575]	##### : 1 [160.48/0.0659]
#0### : 5 [140.73/0.5673]	###1# : 1 [148.55/0.465]	##### : 5 [149.42/0.0913]	##### : 5 [160.04/0.0833]	##### : 0 [218.82/0.0691]
1#### : 1 [146.24/0.0892]	0#### : 2 [152.18/0.3031]	##### : 2 [168.37/0.0824]	0#### : 2 [155.2/0.2989]	##### : 4 [189.27/0.0754]
0#### : 2 [151.85/0.2445]	##### : 5 [156.13/0.0865]	##### : 4 [171.33/0.073]	##### : 2 [182.18/0.0738]	
##### : 1 [150.04/0.0517]	1#### : 5 [152.84/0.0644]	##### : 0 [201.99/0.0622]	1#### : 1 [149.88/0.0426]	
1#### : 5 [158.63/0.0518]	##### : 0 [182.89/0.0547]	0#### : 2 [142.43/0.0701]	1#### : 5 [161.77/0.0406]	
##### : 4 [168.33/0.0764]	##### : 2 [183.1/0.0659]	1#### : 1 [144.33/0.0296]	##### : 0 [185.64/0.0469]	
##### : 0 [201.9/0.0469]	##### : 4 [174.81/0.0721]	##### : 1 [145.13/0.0414]	##### : 1 [153.06/0.0514]	
		##0## : 5 [149.42/0.0581]	####1 : 4 [176.04/0.0623]	
		1#### : 5 [152.96/0.0434]	##### : 4 [176.04/0.0647]	
		###1# : 4 [198.87/0.061]		
		##0## : 1 [145.13/0.0307]		
		##0## : 2 [168.37/0.0419]		
		####1 : 0 [201.99/0.0385]		
		##0## : 0 [201.99/0.0363]		
		####1 : 1 [145.13/0.0279]		
		⋯		

### Discussion

From Table 3 we can see that the LCS approach has worked well on the 4 networks on all but two runs (where the number of rules required was unacceptably high). The overall indication is that *the LCS and rule-compression combination is able to successfully evolve control rules (sets of classifiers) for the full range of networks, requiring fewer than one rule for each state* (with two major exceptions). Performance, in terms of the post-compression rule-set size with minimum number of interventions, ranges from 35 rules to 10 rules (two unusually high outliers also exist, with rule counts of 304 and 168 rules). Performance, in terms of average steps ranges from 4.531 when selecting for minimum number of interventions to a relatively small value of just 2.781. Similarly, the number of average interventions range from 1.714 to just a single intervention on average.

The above results suggest that (1) it is possible to control random Boolean networks with rules developed via learning classifier systems, specifically the ‘standard’ variant of XCS and (2) the ternary structure of the rules tends to enable control of the RBN in question with far fewer rules than there are states in the system.

Figure 11 illustrates the application of a rule set to control Network 4 (shown in Fig. 10). The number under each state string indicates the number of steps required to reach the attractor from that state. The red links represent the application of control rules (the implementation of an action) to intervene in the system. The orange links indicate steps in the system immediately after a control rule has been implemented (please recall that we limit ourselves to a single intervention at once in the current framework and thus a red link is always followed by an orange link). The green links represent steps taken after ‘no action’ (action 0) has been performed.

From consideration of Tables 4, 5, 6 and 7 we can see that there remains considerable variety between rule sets for a particular network instantiation. This suggests that the runs have not managed to converge to the optimal or near optimal solution, given the number of runs and the particular XCS design and parameter settings. This indicates that whilst the LCS successfully evolves control rules, further improvements are possible. Examination of the compressed rule sets indicates the prevalence of ‘overgeneral’ classifiers (i.e. rules of the ##### : action variety) in many of the sets, with a high numerosity relating to these rules. Such rules are likely to lead to a payoff some of the time, whilst producing poor quality results the remainder of the time. The prevalence of these rules indicates that our XCS variant is, in its present form, currently susceptible to the difficulties presented by long ‘action chains’ (Barry 2002). It is also possible that the great many inferior but possible solutions to the control problem also pose a difficulty for the XCS system. It should be noted that *whilst over-general classifiers exist, this does not prevent XCS from successfully controlling the system*.

Training times range from 22–120 s (using an i7-7700HQ processor). On Network 1 training only takes between 22 and 24 s. On Network 2, training takes between 113 and 120 s. On Network 3 training takes 54–57 s. Finally, on Network 4 training takes 63–67 s. This discrepancy in training time indicates that not all networks are equally easy to learn to control. As was recognised earlier and can be seen in Fig. 7, Network 1 is the simplest network to control, and thus it comes as no surprise that it takes the shortest training time. It is interesting that Network 2 takes the longest training time and further investigation in to what constitutes a ‘difficult’ network should be conducted.

The rule sets of 304 and 168 rules represent a puzzle to solve. Initial consideration of the parameters provides an indication of what may have happened. The *ε*_0_ parameter is set to 18.5 whilst the *θ*_*sub*_ parameter is set to 31.579. These high settings both act to prevent inexperienced rules from being subsumed too soon by over-general classifiers. However, it is possible that, as a side effect, this mechanism may be somewhat too effective at preventing subsumption, thus leading to excess rule variety. This is clearly another area where further work is required, to better understand this occurrence.

### Limitations

There are a number of limitations to the study that also serve as opportunities for future work. Broadly these can be listed as follows:

- the overgeneralised classifier problem
- avoidance of exhaustive search for state mapping and compression
- relaxation of the single-intervention-at-once restriction
- modification of the LCS to ensure full state space coverage
- specific nature of the rule set evolved

**Fig. 12:**
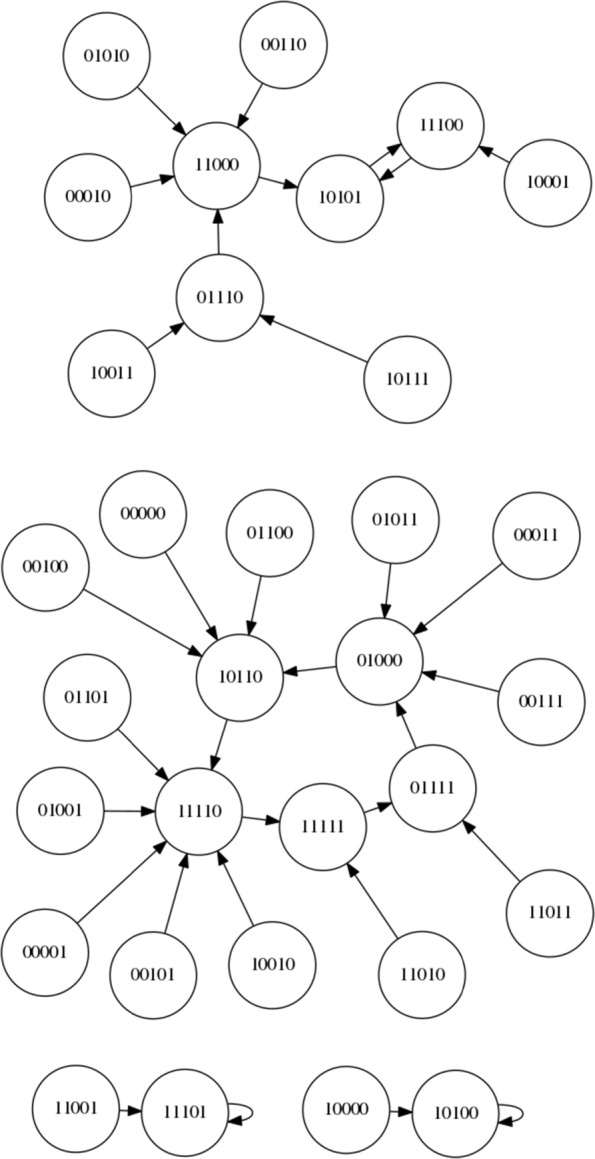
NK Boolean network, instantiation 6 (*N*=5,*K*=2)

The overgeneralised classifier problem raised in “Results and Discussion” Section presents the most pressing area for future work. Some initial work has been done in this area by others and possible modifications to the ‘standard’ XCS system exist that are able to increase the length of the action chains that the system can evolve to navigate. Related to this area is further work involving the development of new XCS variants that can control much larger or more complex RBNs with longer required ‘action chains’. An example of an RBN that is problematic to the current system is presented in Fig. 12. It is possible that the large state cycles in such networks are difficult for the current XCS system to learn – further enhancements may be required for such networks.

Avoidance of exhaustive search is also important for techniques to control larger and more complex systems. In the present system, the original mapping of the state space to locate attractors is done in an exhaustive manner (every initial start point is considered). Exhaustive iteration through start locations is also used in the final stage, both to test that the rule set covers every possible situation.

A relaxation of the single-intervention-at-once restriction could be considered. Presently multiple actions in succession, or an action that flips multiple bits is not considered. It may be that multiple simultaneous interventions are required when operating on more complex networks. Multiple actions could be considered either through converting the action to a bit string such as 10100110 : 00010010 (i.e. flip bits 3 and 6) or by permitting multiple rules to fire at each time instance.

It could be possible to modify LCS to ensure that the rules developed always have full coverage of the state space. This would ensure that the final set of rules produced would cover all possible states in the state space. In contrast, the present implementation sometimes has to prolong the run if the coverage of the state space is incomplete.

A final limitation concerns the specific nature of the rule sets evolved. XCS is able to specifically tailor rules to control a specific network. This may be seen as a strength, because a more general control system may perform less well across all networks. However, this must also be recognised as a drawback of the approach – time must be spent evolving a specific rule set for each target network.

### Future work

There are also a number of future work opportunities that are not related to limitations of the present work:

- new XCS variants that can control more complex RBNs
- application of LCS to control of more realistic or complex network models
- further performance refinements to the current approach
- consideration of situations with limited control nodes
- development of more formal approaches for comparative purposes
- possibilities for control of dynamic networks
- improved integration with existing theory on controllability
- controlling networks with incomplete information

A major opportunity for future work is the application of LCS to more realistic or complex models. The Boolean networks listed by Kim et al. (2013) and Gates et al. (Gates and Rocha 2016) are prime examples of such networks. Work on considering NK Boolean networks with different topologies has been done by Aldana (2003) and control experiments could be performed on such networks. Probabilistic RBNs exist and would present a more challenging control task (Datta et al. 2003).

It would also be possible to adapt existing extensions of XCS (XCSI (Wilson 2001) for integer-based rule conditions and XCSR (Wilson 2000a) for real-valued rule conditions) to control networks with integer-valued or real-valued variables. For example, (Moschoyiannis et al. 2016) performs causal structure analysis on a real world complex network of an industrial ecosystem at the Humber region in the UK, in which the policy decision-making process involves industrial, local government, and NGO (non-governmental organisation) stakeholders. The *factors* (nodes) come with variables that capture controllability and importance. These are attributed with *high*, *medium* and *low* values. Similarly, Schoenenberger and Tanase (2018) consider the Worlds2 system dynamics model. XCS could be used to complement their approach. (Another example where it can be used is to learn customer preferences and make associated recommendations for onward journeys (Matthew R Karlsen and Sotiris Moschoyiannis: Learning condition action rules for personalised journey recommendations, forthcoming)).

Specifically on the subject of asynchronously updated RBNs, further work is also possible. The state spaces of asynchronous RBNs contain more directed links than their synchronously-updated counterparts. As a result, the rule population size *R* would need to be much higher when learning to control these networks. Additionally, the technique (i.e. the application of XCS to controlling RBNs) would require knowledge of which node was about to update so that rules could be related to particular nodes updating (the controller may need to trigger an action if the system is about to unfold in one direction, but not in another). This would require an integer-valued detector indicating the next node about to update. (Alternatively, a probabilistic approach could be adopted.) Something akin to the ‘class imbalance problem’ faced by ‘regular’ (i.e. single-step) classifiers (Hoens et al. 2012) may be encountered since some system states are likely to be extremely rare. This may require one or more adjustments in line with those in the literature. See, for example, the work of Orriols-Puig et al. (2009).

Opportunities for further performance refinements exist, aiming at either minimising the number of interventions required, steps required, or at producing the most compact rule set possible. There are many parameters settings possible in the XCS learning classifier system alone. Additionally, there are many LCS variants to consider. This makes the ‘design space’ (Dennett 1995) of all possible LCS systems very large and thus substantial work remains to explore this space. Furthermore, a similar challenge presents itself in improving the sorting and compression of the rule set at the end of the process. One immediate possible experiment would be to apply the improved deletion scheme suggested by Kovacs (1999).

Herein we have not considered situations in which access to control nodes is limited (i.e. only some subset of the total node set can be controlled) as considered in other approaches (Cornelius et al. 2013). One piece of further work would be consideration of such situations.

Development of more formal approaches for comparative purposes could prove useful. This may be an approach of the kind used by Schoenenberger and Tanase (2018). Alternatively, if a directed graph is produced from one of the original network graphs and then all those states that differ by just one node value are also connected, then a shortest path algorithm could be run to work out the shortest path for each node to a specified attractor. It would be possible to find the route that minimised the number of interventions, in addition to the shortest path. This information could then be used to benchmark the LCS against an ‘optimal’ set of rules. (Note: optimal in terms of actions or steps, not in terms of the size of the rule population.)

Possibilities for control of dynamic networks could also be considered. Savvopoulos et al. (2017) have looked at addressing changes in the topology of a complex network, focusing on random directed graphs, and have also proposed a classification of the nodes based on their impact on the set of control (driver) nodes of the network (Savvopoulos and Moschoyiannis 2017). Other work in this respect includes that of Jia et al. (2013) and Vinayagam et al. (2016) who also focus on changes in the topology in relation to controllability. The classification proposed by Savvopoulos and Moschoyiannis (2017) in fact draws from these works. Learning classifier systems are able to adjust to changing environments. An LCS could be constructed such that it adapts to the addition and removal of nodes or links of the network, or to changes in the way each node processes information. The latter can be exploited in more general intelligent digital ecosystem architectures (Marinos *et al*^2011^) where the processing at each node may involve the execution of micro-services and long-running transactions (Marinos et al. 2009*;*Moschoyiannis and Krause 2015).

The XCS system does not require any knowledge of the system under control other than that available via the detectors. Indeed, the condition–action rules evolve from scratch to model the external system. However, there may be circumstances where only a subset of detectors are available and we wish to control one or more of the variables covered by the detectors without being aware of the *full* state of the system. It may be that XCSI is able to ‘bridge’ the unknowns here and continue to control the system. Such an investigation marks an important piece of future work.

On a related note, it may be possible that the network model that XCS learns on is incomplete or has inconsistencies or errors in it. This is an especially challenging area for the application of XCS (or, indeed, other control methods). Missing model components must be completed before XCS is run on the model. For instance, missing Boolean functions on a non-random Boolean network modelling a real-world system would have to be filled in. This can be done in a random manner, producing a range of possible network structures. XCS can then be run on these structures, producing a range of possible controllers. Data on the real world network can then be used to eliminate erroneous network structures and associated controllers until a useful controller remains. This is not dissimilar to the approach within the work of Giacomantonio and Goodhill (2010) but for the evolution of a controller. Approaches designed for XCS to cope with noise or missing information are covered in a review by Wilson (Wilson 1999) and may also be helpful.

A further line of work is achieving greater integration between notions of controllability when considering pure network structures and notions of controllability when considering the dynamics of networks. In the traditional sense a network is controllable when it can travel from one state to any other state in finite time (Moschoyiannis et al. 2016). Here we consider a network to be controllable *with respect to a particular set of control nodes and a particular goal state or states* if the desired state can be reached from any other state in finite time. *Controllability with respect to every attractor could well be achieved by providing a detector and associated classifier condition for desired attractor state.* In this way, a subset of rules would evolve for each targeted attractor, though the rule sets would still overlap where possible.

## Related work

Previous work on NK Boolean networks was completed by Kauffman (1969*,*^1989^*,*^1993^) and collaborators, with later refinements. The work is important in relation to understanding gene regulatory networks and other types of biological networks as considered by Kim et al. (2013). Work on the control of complex networks (with associated concepts such as basin of attraction, state cycle, attractor, and so on) has been carried out by a number of authors (Cornelius et al. 2013*;*Kim et al. 2013*;*Li et al. 2015*;*Zañudo and Albert 2015*;*Gates and Rocha 2016*;*Hou et al. 2016). Understanding how to control complex networks could assist in interventions in to the aforementioned biological networks or in to artificial networks such as power grids, as considered by Cornelius et al. (2013).

Work on the XCS variant of learning classifier systems was originally completed by Wilson (1995*,*^1998^). XCS departs from previous LCS designs by judging the performance of rules according to their predictive accuracy rather than their payoff, and by applying the genetic algorithm to the action set rather than the rule population as a whole (Urbanowicz and Moore 2009). Urbanowicz and Moore (2009) recognise XCS as one of the most popular LCS systems. The success of XCS is likely due to the improved performance resulting from the design changes implemented.

Work *has* previously been completed in the intersection between random Boolean networks and learning classifier systems (Bull 2009*;*Preen and Bull 2009). *However*, this previous work focuses on using Boolean networks as an alternative to the conventional string based condition-action rules, whilst studying ‘multiplexer’ and ‘maze’ tasks rather than on studying interventions in the control of complex networks. (See also the earlier work of Forrest and Miller (1990) which demonstrates the relationship between LCS rules and Boolean networks.) The Boolean multiplexer task is a single-step task and thus does not require action chains. The maze task is similar to the problem considered herein but differs in that the state of the maze is static unless the animat agent makes a move, whilst the networks herein unfold according to their own logic even without the intervention of the LCS.

## Concluding remarks

We have described an approach to evolving rule sets that can steer a complex network towards a desired state. The set of ‘control rules’ evolves over time to reflect the structure and dynamics of the network in question. The structure and dynamics of networks have been studied in a number of domains, including biology (Kauffman 1993) (e.g., gene regulatory networks, cell signalling networks (Kim et al. 2013) or protein interaction networks (Jia et al. 2013)), policy-making (e.g., transition of a region from a fossil fuel to a bio-based economy (Moschoyiannis et al. 2016)), social network analysis (Gaito et al. 2012), economics (Anderson et al. 1988), transport (e.g., networks of commuter journeys in). Our approach advocates the application of learning classifier systems, specifically the XCS variant (Wilson 1995*;*^1998^), to control random Boolean networks of the NK type (Kauffman 1993).

Learning classifier systems are programs that learn to respond in a useful way to environmental stimuli, combining both evolutionary algorithm and reinforcement learning components (Urbanowicz and Moore 2009). Random Boolean networks exhibit complex behaviour with large state space, many possible trajectories through this space, and multiple basins of attraction. The precise graph topology of a random boolean network is determined by the exact network constructed and examined. The LCS was run on 4 different Boolean network structures (5 times on each network). We have shown that it is possible to learn rules to control complex networks through the application of an LCS.

LCS rules are ternary in nature, providing a compact rule structure with fewer rules than there are states in the system. The *best* performing rule sets evolved for four 32-state random Boolean networks require an average number of interventions ranging from 1 per trajectory to 1.714 per trajectory (where a trajectory starts from a random point in the state space and ends at a desired attractor state) and require between 10 to 18 rules to achieve this (depending on network complexity). We stress that the LCS learns to control the system instances without human intervention and that both the structure and dynamics of the network are automatically taken in to consideration whilst the rule set evolves.

Immediate future work to overcome the limitations of this work includes the development of pre-processing and post-processing procedures that do not require exhaustive search, modification of XCS to avoid overgeneralised rules and further modifications to ensure full rule coverage of the state space for every complete run. The most promising future work involves investigating the control response in dynamic networks and consideration of constrained control situations where only a subset of nodes are controllable.

## Abbreviations

AI: The average number of interventions
AS: The average number of steps required to reach the attractor with a given rule set
CR: Compressed rules
GA: Genetic Algorithm
GRN: Gene regulatory network
LCS: A learning classifier system
Net: Network instance number
NGO: Non-governmental organisation
RBN: Random Boolean Network
RP: Reinforcement program
XCS: eXtended Classifier System
XCSI: eXtended Classifier System (Integer-valued)
XCSR: eXtended Classifier System (Real-valued)
XOR: eXclusive OR
